# Knowledge and exposure to complementary and alternative medicine in paediatric doctors: a questionnaire survey

**DOI:** 10.1186/1472-6882-7-38

**Published:** 2007-11-29

**Authors:** Simon Fountain-Polley, Grace Kawai, Amanda Goldstein, Titus Ninan

**Affiliations:** 1 Department of General Paediatrics, Birmingham Heartlands Hospital, Bordesley Green East, Birmingham, B9 5SS, UK; 2 Birmingham Children's Hospital, Steelhouse Lane, Birmingham, B4 6 NH, UK

## Abstract

**Background:**

Complementary and alternative medicines are increasingly used by the general population. A survey was conducted to ascertain the knowledge of Complementary and Alternative Medicines (CAMs) amongst paediatric physicians, and whether seniority increases the likelihood of its use being considered in consultations, or of families discussing it.

**Methods:**

Anonymous survey of general paediatric doctors in a large inner-city district general hospital (DGH) and tertiary children's centre (TC) using a questionnaire. Statistical analysis was calculated using Minitab.

**Results:**

43/49 (88%) questionnaires were returned correctly. 13 (30%, CI 17 – 46%) doctors had personally used CAMs. 24 (56%, CI 40 – 71%) of their families had used CAMs. 13 (30%, CI 17 – 46%) had received formal CAMs education. 21 (49%, CI 40 – 71%) could name a total of 5 types of CAMs. Consultants were significantly more likely to ask about CAM use than middle-grades and juniors (p < 0.05, CI 48 – 93%, 35 – 90%, 8 – 33% respectively) and have had a clinical encounter where they felt it was significant. 32 (74%, CI 59 – 86%) of the clinicians had been asked about CAMs. 33 (77%, CI 61 – 88%) of doctors had successful CAM use reported to them, and 20 (47%, CI 31 – 62%) had failure of CAMs reported to them.

**Conclusion:**

CAM use is relatively common in paediatric doctors and their families. They have received little formal CAMs education. Consultants were more likely than juniors to ask about CAM use and have had a clinical encounter where it played a significant part. Around half of all doctors irrespective of grade have been asked about CAMs in a clinical encounter.

## Background

Over recent years Complementary and Alternative Medicines (CAM) have received increasing attention in medical and media circles. Despite a recent central government report in the UK suggesting that the use of CAMs requires further research and regulation [1], patients and their families continue to use them. A recent survey found that around 10% of adults in the United Kingdom had used CAM in the preceding year [2]. The usage amongst children has been estimated between 1.8 – 17.9% in the general population [3,4], and 11 – 51% in hospital outpatient settings [5,6]. Despite the large number of parents allowing CAM usage for their children, only 6% of paediatricians ask about it, in contrast to 53% of parents expressing a desire to explore CAM use [7]. Our aim was to elucidate the numbers of paediatric doctors asking about CAM use in 2 Birmingham hospitals, and to collect data on their knowledge and exposure to CAMs. We hypothesised that senior clinicians may be more likely to specifically ask or have interacted with patients on CAMs.

## Methods

**Figure 1:**
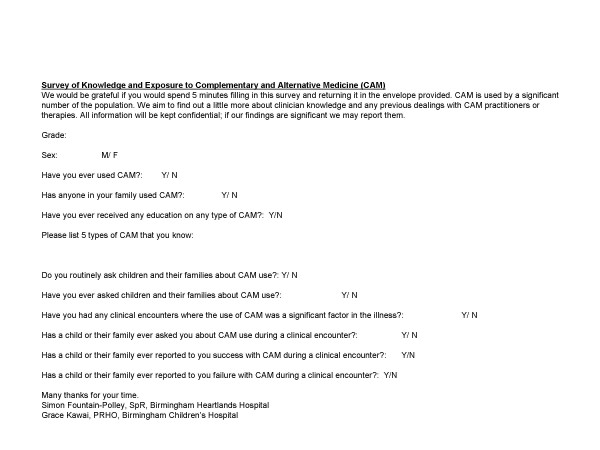
Questionnaire Distributed to medical staff.

We devised an anonymous questionnaire to elicit any CAM use by individual clinicians and their family, and to name 5 different CAMs. The rest of the questionnaire focused on whether clinicians enquired into patient CAM use, and whether parents had communicated to their clinician a desire to discuss it (see Figure 1). The questionnaire was designed to record the respondents' gender and seniority, and was provided with an envelope for return to the data collectors. The questionnaire was distributed to all the general paediatric doctors in a large district general hospital (DGH = Birmingham Heartlands Hospital, n = 29), and the general paediatric team in the tertiary centre children's hospital (TC = Birmingham Children's Hospital, n = 20).

The doctors were grouped into consultants, middle-grades (doctors acting at registrar level), and juniors (SHOs/FY2, and FY1). In total, questionnaires were distributed to 18 consultants (DGH = 10, TC = 8), 14 middle-grades (DGH = 9, TC = 5), 14 SHOs/FY2s (DGH = 8, TC = 6), and 3 FY1s (DGH = 2, TC = 1).

Both hospitals serve a diverse ethnic population, and provide general paediatric inpatient and outpatient facilities. Statistical analysis was completed with Minitab using either Fisher's Exact Test or chi-squared/chi-squared for trend for comparisons across the grades.

## Results

The overall response rate was 43/49 (88%). One questionnaire was returned but incompletely answered so excluded from the data. The response rate was 26/29 (90%) from the DGH, and 17/20 for the tertiary centre (85%). Table 1 contains the answers to the demographic questions and personal clinician use to CAMs grouped by gender. Personal use of CAMs (30%, CI 17 – 46%) was less than reported use by family members. Around a third of respondents reported some formal education in CAMs. These included all the FY1 doctors and 22% of the consultants. Only 14% of the middle-grades had received any education on CAMs. Receiving formal education on CAMs did not lead to a significant reported routine enquiry into CAM use (FET, p = 1.00).

**Table 1:** CAM Exposure related to gender

	Male Number (%)	Female Number (%)	Total Number (%) Confidence Interval
Personally used CAM	5 (26)	8 (33)	13 (30) 17 – 46%
Family used CAM	13 (68)	11 (46)	24 (56) 40 – 71%
CAM Education	5 (26)	8 (33)	13 (30) 17 – 46

Of all the respondents 56% (CI 40 – 71%) could name 5 types of CAMs, with only 1 person unable to name any. The 5 most named were: acupuncture (34), homeopathy (27), ayurvedic medicine (17), aromatherapy (15), and herbal medicines (13).

Table 2 records the clinician-parent interaction with CAMs, categorised by seniority. The SHO's, FY2, and FY1 doctors were grouped together for analysis of the results.

**Table 2:** Clinical Encounters with CAMs compared to Seniority

	Consultant Number (%)	Mid-grade Number (%)	Juniors Number (%)	P value Test
Routinely ask about CAMs	5 (31)	1 (8)	0 (0)	<0.05 FET
Ever asked about CAM use	12 (75)	8 (67)	4 (27)	<0.01 Chi-squared for trend
Clinical encounter where CAM significant	11 (69)	3 (25)	2 (13)	<0.01 Chi-squared for trend
Ever been asked about CAM	14 (88)	9 (75)	9 (60)	NS FET
Success reported with CAM	15 (94)	9 (75)	9 (60)	NS FET
Failure reported with CAM	11 (69)	4 (33)	5 (33)	<0.05 Chi-squared for trend

Consultants were significantly more likely to report routinely asking about CAM use (p < 0.05, FET). When comparing the grades with regards to reportedly ever asking about CAM use, there was a significant difference through the grades, with consultants (75%, CI 48 – 93%) being more likely than middle-grades (67%, CI 35 – 90%), who were more likely than juniors (27%, CI 8 – 33%) to have done so (chi-squared for trend = 7.08, p < 0.01). The same was true for clinicians reporting an encounter where CAM use was significant over the various grades (chi-squared for trend = 10.05, p < 0.01). No statistically significant trends were noted for clinicians across the grades being more likely than others to have been asked about CAM use by families, or to have success with CAMs reported to them. Interestingly, consultants were more likely to have had failure with CAMs reported to them (chi-squared for trend = 3.883, p < 0.05).

Personal use, or family use of CAMs was not significantly associated with reported routine enquiries about its use (FET, p = 0.345, p = 0.68 respectively). These results were not affected by gender or hospital setting (tertiary or district general hospital).

## Discussion

Our survey aimed to examine the knowledge and exposure of paediatric doctors to CAMs in their lives and daily practice. Around a third of respondents had used CAMs themselves, which is greater than expected, and higher than the national average use (10%) [2]. Usage was not affected by gender. Reported CAMs use by the clinicians' family was greater than personal use, and much higher than the general population of the UK. Personal or family use did not translate into an increased likelihood of reportedly routinely asking about CAM use in patients, suggesting that the respondent's own health beliefs do not necessarily reflect their practice. This contrasts with a survey of American physicians that found they recommended CAMS more if they themselves had used it [8].

A small proportion of respondents reported formal education in CAMs, although the questionnaire did not elicit the exact nature of this. In view of public interest, this is surprising, as training should reflect current trends in illness behaviour. All the FY1 doctors had received some teaching on CAMS, which may signal changing medical school curricula. Undergraduate teaching in CAMs requires integration into the entire curriculum and is problematic [9]. Even when introduced it is taught with diverse formats and mainly as a broad introduction to the subject [10]. A previous survey of paediatrician's attitudes towards CAMs found 54.1% of them were interested in further education on it [11], and our respondents may be expected to have a similar desire. A survey of American physicians concluded education on CAMs represents an unmet need in their respondents [8].

The most commonly named CAMs were similar to those used by the general population [2], with the exception of osteopathy and chiropractic. For children, the respondents named similar treatments to those most used in the UK [4] (homeopathy, aromatherapy, herbal medicines, osteopathy, and reflexology), with the exception of acupuncture and ayurvedic medicine. This may reflect an adult perspective on CAMs, or differences in the ethnic background of the respondents. This contrasts with an American survey where 55% of respondents had never heard of ayurvedic medicine [12]. Individuals' knowledge may be related to cultural and institutional awareness or availability of CAMs. Throughout the world different countries and cultures have differing alternative therapies. Where a clinician lived and trained may affect their attitude and acceptance towards other therapies, and also their knowledge of them. Medical workforce mobility in the UK may explain the high recall of ayurvedic medicine.

A clear trend was noted across the grades with regards to reportedly routinely asking about CAM use. Senior doctors were more likely to have communicated over CAM use, which may reflect increased experience. A total of 31% of the consultants routinely asked about CAM use; a total of 14% of doctors working in paediatrics asked. This is more than the 6% of parents who reported their paediatrician asking about CAM use in the USA [7] (including 348 surveys, 92% response rate). In our survey, 76% of all respondents had been asked about CAM use, including 88% of the consultants. Alternative therapies seem to be commonly considered in the treatment of childhood ailments.

The majority of paediatricians had CAMs success, and just under half, had CAMs failure reported to them. These outcomes indicate the relevance of systematically asking patients' families about CAM use. Full disclosure of alternative treatments may prevent undesirable interactions or failure of conventional treatments. An atmosphere to ensure this does not occur should be encouraged.

All of the paediatric doctors surveyed were senior general paediatricians or doctors rotating through general paediatrics. It would be interesting to assess the knowledge and encounters with CAMs of other members of the paediatric community based in the various subspecialties by repeating the questionnaire. To our knowledge this has not been done despite the widespread use of CAMs in children with cancer, dermatological disorders, juvenile arthritis and asthma [13].

The survey population was relatively small; however the response rate was excellent, which may reflect general interest in the CAM topic amongst paediatricians. Recall bias may have affected results. However, as CAMs is perceived as a controversial and uncommon topic, we anticipated that consults including CAM discussions would be well remembered. CAMs were not specifically defined so that physicians' responses were not biased or directed in any way.

## Conclusion

As the use of CAMs is relatively common amongst the population, paediatric doctors should be routinely enquiring into its use. As expected, the more senior doctors were more likely to routinely ask about CAM use, and have had a clinical encounter where CAM use had played a significant part. Formal education in CAMs is low amongst doctors working in paediatrics in Birmingham, especially amongst specialist trainees. Integrating CAM education into the medical curricula for medical students, and continuing education in postgraduates, are areas for further research and development.

## Competing interests

The author(s) declare that they have no competing interests.

## Authors' contributions

SFP co-wrote the questionnaire, distributed it, collated the data, analyzed the data, and drafted the manuscript. GK co-wrote the questionnaire, distributed it, collated the data, analyzed the data, and helped draft the manuscript. AG revised the questionnaire and manuscript and helped interpret the data. TN revised the questionnaire and manuscript and helped interpret the data. All authors have read and approved the final manuscript.

## Pre-publication history

The pre-publication history for this paper can be accessed here:


